# Ultrasound-Guided Diagnosis and Percutaneous Drainage in Subacute Necrotizing Fasciitis of the Lower Extremities: A Report of Two Cases

**DOI:** 10.7759/cureus.76541

**Published:** 2024-12-28

**Authors:** Shingo Sasamatsu, Toru Kanno, Satoshi Yoshikawa, Takeshi Ueda

**Affiliations:** 1 Emergency and General Internal Medicine, Rakuwakai Marutamachi Hospital, Kyoto, JPN; 2 Radiology, Dokkyo Medical University, Mibu, JPN

**Keywords:** drainage, necrotizing fasciitis, necrotizing soft-tissue infection, staphylococcus aureus, ultrasonography

## Abstract

Necrotizing fasciitis (NF) is a life-threatening disease that is diagnosed through an exploratory incision and typically requires surgical debridement. Reports of non-surgical cures are limited to specific cases, such as NF affecting only the head and neck regions. The two patients (a woman and a man) were both in their 70s and underwent maintenance dialysis for diabetic nephropathy. Both presented with leg pain. Samples were obtained using ultrasound-guided aspiration, and NF was diagnosed based on the characteristics (dishwater-like gray exudate) of the samples and the presence of gram-positive cocci (the culture results identified *Staphylococcus aureus*). Although an initial treatment with antimicrobial agents was administered, no improvement was observed. Percutaneous drainage was then performed, which led to successful outcomes in both cases. In addition to the clinical course, blood tests, and imaging studies, ultrasound-guided needle aspiration can aid in the accurate diagnosis of NF. Percutaneous drainage may be a minimally invasive alternative to surgical debridement for subacute NF. Nonetheless, careful consideration should be given to the indications for percutaneous drainage.

## Introduction

Necrotizing soft-tissue infection (NSTI) is characterized by widespread tissue destruction extending from the epidermis to deep musculature. Necrotizing fasciitis (NF) is a subset of NSTI. It is characterized by superficial fascia fragility, dishwater-like gray exudate, and absence of pus and is typically found using exploratory incision [1].

*Streptococcus pyogenes* (group A) is the predominant causative pathogen, followed by *Staphylococcus aureus* [2]. Type II NF, caused by *S. pyogenes*, often follows a time course that is associated with a high mortality rate [3]. Moreover, the mortality rate of NSTI is approximately 20% [4]. According to a review by Nawijn et al., the mortality for NSTI does not significantly decrease within 24 hours of presentation (OR 0.79) but decreases significantly within 12 and six hours (OR 0.43) [4]. Therefore, early surgical debridement after disease onset is crucial to improve patient survival rates. However, we are hesitant about this approach because surgical debridement is invasive, often requires a surgeon's sign-off, and requires general anesthesia.

We report two cases of subacute NF of the lower extremities caused by methicillin-susceptible *S. aureus* (MSSA), which were diagnosed using ultrasound-guided aspiration and Gram staining. These patients were treated using antimicrobial agents and percutaneous drainage without surgical debridement.

## Case presentation

Case 1

A woman in her early 70s on maintenance dialysis for diabetic nephropathy visited our hospital because of left leg pain that had persisted for two days. At the time of initial examination, her temperature was 36.7°C, blood pressure was 68/45 mmHg, pulse rate was 88 bpm, respiratory rate was 29 breaths/minute, and oxygen saturation (SpO2) was 92% (room air). Her Glasgow Coma Scale (GCS) score was E3V5M6 (total GCS score 14/15), and warmth and swelling were noted in her left thigh. No crepitus was observed. The patient had a positive left psoas sign, positive left costovertebral angle tenderness, and positive left obturator sign. No evidence of trauma or redness was observed at the shunt puncture site. The laboratory parameters on admission of the patient are listed in Table 1. Her laboratory risk indicator for NF (LRINEC) score was 7.

**Table 1:** Laboratory parameters of on admission (Case 1)

Parameter	Value	Reference range
White Blood Cell (WBC)	9.1 × 10³/μL	3.5–9.1 × 10³/μL
Hemoglobin (Hb)	11.7 g/dL	11.3–15.2 g/dL
Platelet Count	16.4 × 10⁴/μL	13.0–36.9 × 10⁴/μL
C-Reactive Protein (CRP)	20.2 mg/dL	0.00–0.14 mg/dL
Blood Urea Nitrogen (BUN)	38.5 mg/dL	8.0–20.0 mg/dL
Creatinine	6.62 mg/dL	0.46–0.79 mg/dL
Albumin	2.5 g/dL	3.8–5.2 g/dL
Creatine Kinase (CK)	122 U/L	45–163 U/L
Glucose	177 mg/dL	70–109 mg/dL
Hemoglobin A1c (HbA1c)	6.7%	4.6–6.2%
Lactate	4.9 mmol/L	0.5–2.2 mmol/L

**Figure 1:**
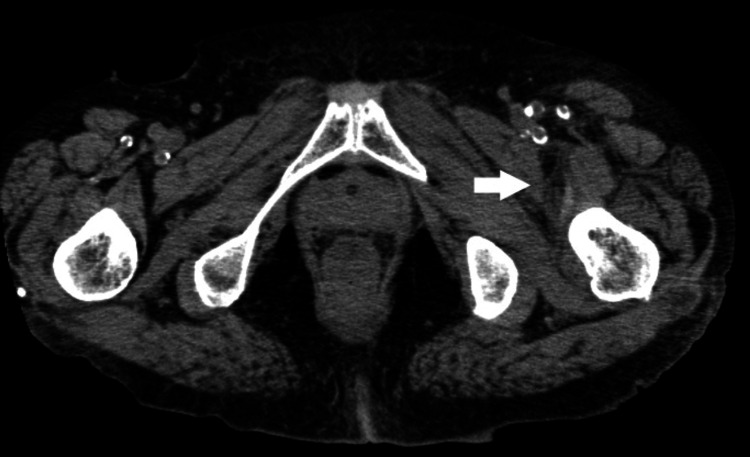
CT of the hip region (Case 1) showing increased lipid concentration and fluid in the anterior aspect of the left thigh (arrow).

**Figure 2:**
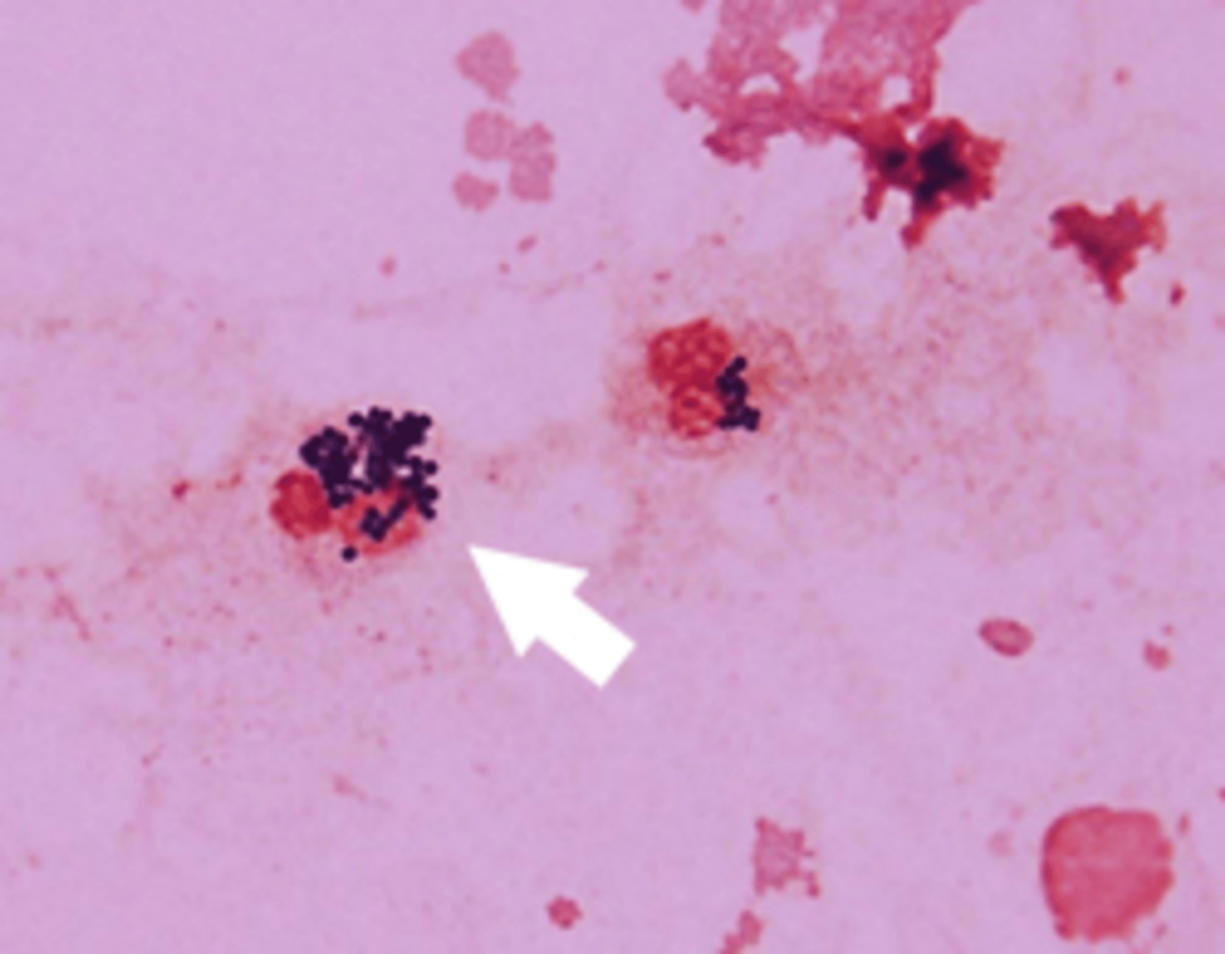
Gram staining (Case 1) of aspiration fluid revealed numerous GPC clusters (arrow). GPC: gram-positive cocci

A computed tomography scan (CT) showed increased periprosthetic fatty tissue density and ventral fluid from the left iliopsoas muscle to the anterior aspect of the left thigh (Figure 1). Ultrasonography showed a 4-mm echo-free space along the fascia. These findings suggested perimuscular inflammation and fluid collection along the fascia. Ultrasound-guided aspiration revealed a dishwater-gray exudate. Gram staining revealed numerous gram-positive cocci (GPC) clusters (Figure 2).

Clinically, the patient was diagnosed with NF. Noradrenaline (NAD) and vasopressin were required to maintain blood pressure. As she was in a state of septic shock, vancomycin (VCM) 1 g/day and meropenem (MEPM) 0.5 g/day were initiated. VCM was intended to cover methicillin-resistant *S. aureus* (MRSA), whereas MEPM provided broad-spectrum coverage against gram-negative and anaerobic bacteria. Following consultation with a surgeon, the CT was interpreted as showing mild swelling of the left iliopsoas muscle along with fluid accumulation on the iliacus muscle. It was concluded that the fluid originated from an iliopsoas abscess and descended into the left anterior thigh. The likelihood of NF was low due to the absence of redness, skin bullae, or necrosis on the skin, combined with the minimal fluid collection observed on CT. Consequently, exploratory incision and surgical debridement were not performed. On day 4, when NAD was completed, the patient's WBC count was 12.8 × 10^3^/µL, c-reactive protein (CRP) was 5.66 mg/dL, and the swelling in her thigh had improved. MSSA was detected in the exudate culture. Cefazolin (CEZ) was selected as a single agent based on its tissue penetration into soft tissues and its stability against beta-lactamase.

**Figure 3:**
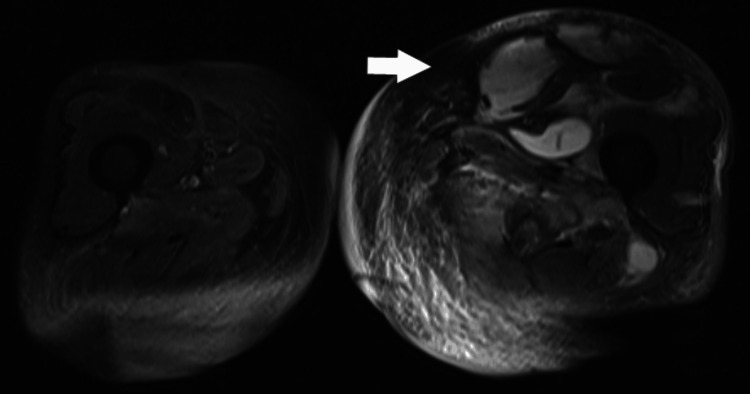
STIR MRI of the thigh (Case 1) showing fluid retention in the anterior aspect of the left thigh (arrow). STIR: short tau inversion recovery

On day 25 of hospitalization, her WBC count improved to 9.0 × 10^3^/μL, and CRP improved to 0.41 mg/dL. However, the swelling in the left thigh persisted. A short tau inversion recovery (STIR) sequence on magnetic resonance imaging (MRI) showed subcutaneous to muscle tissue inflammation and increased fluid retention in the anterior aspect of the left thigh (Figure 3). STIR MRI, with its fat suppression effect, allows for detailed evaluation of inflammation and fluid collection.

A small incision was made directly above the fluid area that revealed a fragile fascia and approximately 150 mL of fluid was drained. After administering local anesthesia with 1% lidocaine, a small incision was made. A 6-mm Nelaton catheter with additional side holes was inserted into the loose connective tissue above the fascia to facilitate drainage. Nelaton catheter is relatively firm among soft tubes, making it easy to insert. The wounds were percutaneously cleaned daily using saline solution. On day 35, the catheter was removed after confirming that drainage had decreased and normalized, and antimicrobial administration was terminated. No recurrence was observed.

Case 2

A man in his early 70s on maintenance dialysis for diabetic nephropathy visited our hospital for left leg pain that had persisted for seven days. At the time of initial examination, his temperature was 36.9°C, blood pressure was 108/62 mmHg, pulse rate was 96 beats/minute, respiratory rate was 15 breaths/minute, oxygen saturation was 95% (room air), and GCS score was E4V5M6 (14/15). Swelling, pale redness, warmth, and severe tenderness on palpation were observed in the left thigh to the lower leg. No trauma scarring or redness was palpable at the puncture site of the arteriovenous fistula. The laboratory parameters of the patient on admission are listed in Table 2. His LRINEC score was 9.

**Table 2:** Laboratory parameters on admission (Case 2)

Parameter	Value	Reference range
White Blood Cell (WBC)	14.7 × 10³/μL	3.9–9.8 × 10³/μL
Hemoglobin (Hb)	11.4 g/dL	13.5–17.6 g/dL
Platelet Count	10.4 × 10⁴/μL	13.1–36.2 × 10⁴/μL
C-Reactive Protein (CRP)	25.8 mg/dL	0.00–0.14 mg/dL
Blood Urea Nitrogen (BUN)	51.8 mg/dL	8.0–20.0 mg/dL
Creatinine	7.84 mg/dL	0.65–1.07 mg/dL
Albumin	2.8 g/dL	3.8–5.2 g/dL
Creatine Kinase (CK)	18 U/L	59–248 U/L
Glucose	165 mg/dL	70–109 mg/dL
Hemoglobin A1c (HbA1c)	6.6%	4.6–6.2%
Lactate	2.0 mmol/L	0.5–2.2 mmol/L

**Figure 4:**
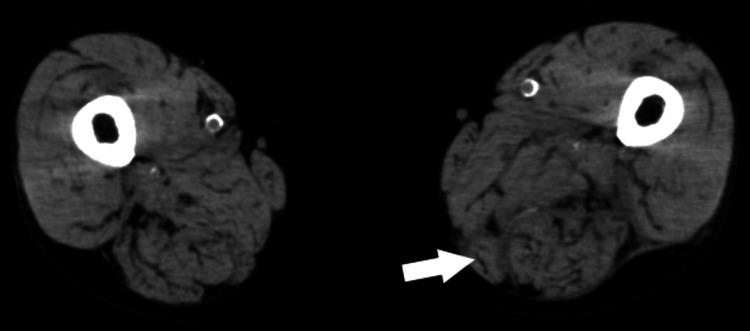
CT of the thigh (Case 2) showing myxomatosis of the medial thigh to the posterior thigh compartment (arrow).

CT showed myxomatosis from the medial thigh to the posterior thigh compartment (Figure 4). These findings suggested the presence of soft tissue inflammation. However, it is unclear whether this represents the fluid collection at this stage. 

Owing to the subacute course and the patient’s generally good condition, we concluded that there was little reason to actively suspect NF and perform an exploratory incision. Furthermore, VCM was administered to treat potential NF. We also did not rule out the possibility of pyomyositis or diabetic muscle infarction. On day 6, his pain improved, his WBC count decreased to 10.1 × 10^3^/μL, and his blood culture was negative.

**Figure 5:**
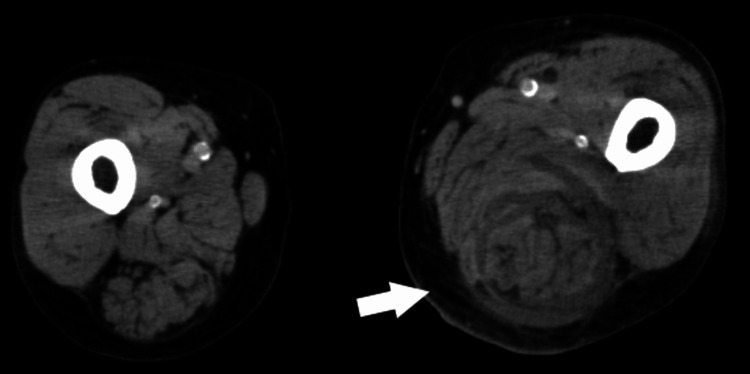
CT of the thigh (Case 2) showing fluid accumulation on the fascia of the posterior femoral compartment (arrow).

**Figure 6:**
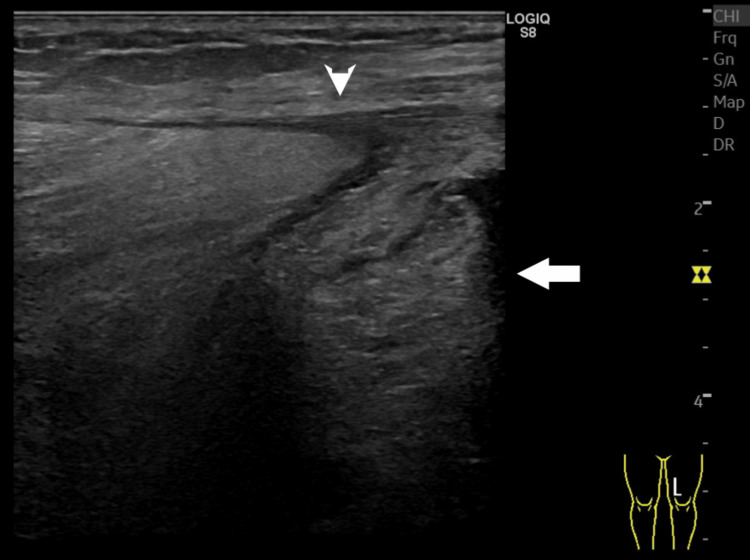
Ultrasonography of the left thigh (Case 2) showing fluid accumulation (3 mm; arrowhead) and myxomatosis (arrow) on the fascia.

On day 8, his pain increased and redness was observed on his left thigh. His WBC level was elevated to 20.4 × 10^3^/μL. CT revealed worsening myxomatosis and a small amount of fluid accumulation from the medial posterior aspect of the thigh to the lower leg (Figure 5). Ultrasonography showed a 3-mm echo-free space along the fascia (Figure 6). We suspected this fluid over the muscles indicated NF. Ultrasound-guided aspiration revealed a dishwater-gray exudate and Gram staining showed numerous GPC clusters.

**Figure 7:**
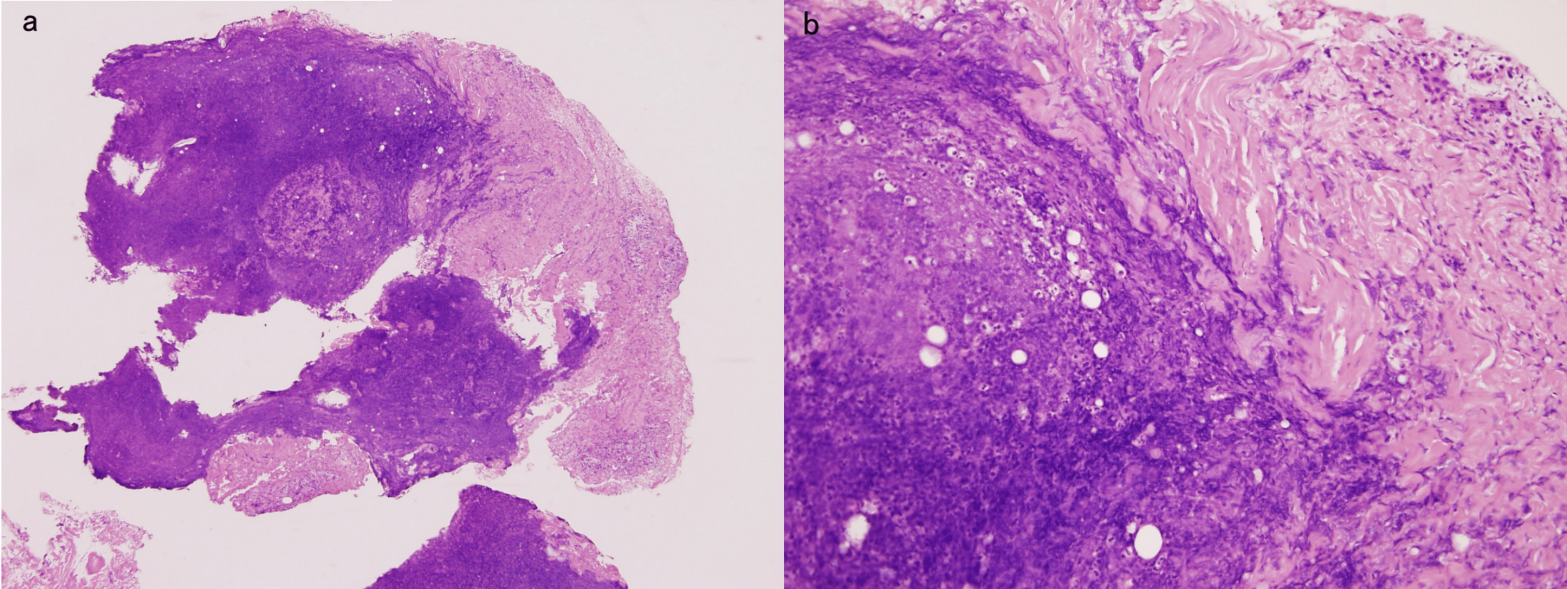
Histopathology (Case 2) of the fascia (hematoxylin and eosin stain, a: 40×. b: 200×); (a) necrotic tissue and (b) fibrous connective tissue with inflammation.

Surgical debridement was proposed which was declined by the patient due to the invasive nature of the procedure. Considering the sub-acute progression and his relatively stable general condition, we decided on percutaneous drainage. A small incision was made, and internal observation revealed a fragile fascia that was clinically diagnosed as NF. The fascia appeared relatively preserved. A biopsy of the fascia was taken for pathological examination. Using the same procedure as in Case 1, a 6-mm Nelaton catheter was percutaneously inserted into the coarse tissue above the fascia in the posterior region of the left thigh and the posterior region of the lower leg to facilitate drainage. The wounds were cleaned daily through the catheter using a saline solution. On day 13, MSSA was detected in the culture of the exudate, and CEZ was administered. The patient’s subjective symptoms, local findings, WBC count, and CRP levels improved thereafter. On day 21, we confirmed that the drainage had decreased and normalized, the catheter was removed, and antimicrobial therapy was terminated. No recurrence was observed. Pathological examination revealed necrotic tissue and inflamed fibrous connective tissue (Figure 7). Findings of fascial inflammation and necrosis supported the clinical diagnosis of NF.

## Discussion

Many patients with NF often have underlying medical conditions. Patients with diabetes mellitus (DM) tend toward vascular disease, peripheral neuropathy, delayed skin wound healing, and susceptibility to infection. Risk factors for NF of the lower extremities include obesity, nicotine addiction, use of non-steroidal anti-inflammatory drugs (NSAIDs), spontaneous cosmetic bleaching, and DM [5]. As observed in our second case, NSAIDs and other sedative drugs may reduce fever and pain and delay the diagnosis.

Diagnosis of NF is challenging. Its early presentation often mimics the clinical course of cellulitis [6]. Studies have reported that only 15-34% of patients with NF have an accurate diagnosis [2,7]. Several clinical and imaging findings have been evaluated. The sensitivity and specificity of clinical findings were 46% and 77%, respectively, for fever, 25.2% and 95.8%, respectively, for hemorrhagic bullae, and 21% and 97.7%, respectively, for hypotension. The sensitivity and specificity of imaging findings were 48.9% and 94%, respectively, for the visualization of soft tissue gas on plain radiography, 85.5% and 93.3%, respectively, for the visualization of fascial gas on CT, and 94.3% and 76.6%, respectively, for the presence of fascial enhancement, fascial edema, or fascial gas on CT.

The LRINEC score has been a subject of debate [8]. It is based on laboratory indicators including WBC, hemoglobin, sodium, glucose, creatinine, and CRP. It has been reported that a score of ≥6 demonstrates a sensitivity and specificity of 68.2% and 84.8%, respectively, whereas a score of ≥8 shows a sensitivity and specificity of 40.8% and 94.9%, respectively [9]. Consequently, it is considered unreliable in ruling out NF. The modified LRINEC score incorporates lactate and liver disease as additional parameters. It has been reported to achieve the sensitivity and specificity of 91.8% and 88.4%, respectively [10]. However, the effectiveness of this new scoring system warrants further investigation. The ultrasonographic findings of NF include (i) fluid accumulation along the fascia, (ii) irregular fascia, and (iii) diffuse fascial thickening, which is not typically observed in cases of cellulitis [11]. In a study by Yen et al., when "diffuse thickening of subcutaneous tissue with a fluid accumulation of 4 mm or more along the fascia" was defined as a finding, its sensitivity, and specificity were 88.2% and 93.3%, respectively [12]. The sensitivity and specificity were 75% and 70.2%, respectively, when the cut-off for fluid retention was set to 2 mm [13].

Despite leveraging the clinical course, physical findings, LRINEC score, and imaging studies, significant limitations remain in the clinical diagnosis of NF. Thus, when NF is suspected, an exploratory incision is required [9]. However, exploratory incision is invasive, often necessitates consultation with specialized departments, and is not a readily accessible diagnostic option for internists to perform promptly. In contrast, ultrasound-guided aspiration is a less invasive alternative and may serve as a useful diagnostic tool. Additionally, ultrasound-guided aspiration can be used to collect specimens from fluid reservoirs of a few millimeters. In these cases, drainage and Gram staining findings from ultrasound-guided aspiration can be used as diagnostic aids. There are no reports on the sensitivity and specificity of combining ultrasound findings, drainage findings, and Gram staining results. However, integrating these modalities is expected to further enhance diagnostic accuracy. The threshold for performing aspiration is likely lower than that for exploratory incision. However, aspiration has limitations, including the inability to directly observe the tissue, the lack of tactile feedback, and the possibility of obtaining only a small sample. 

As in Case 2, early diagnosis of NF without a typical clinical course is challenging. If ultrasound-guided needle aspiration had been performed at that time, a better clinical course could have been expected. To prevent diagnostic delays, we propose proactively performing aspiration whenever NF is clinically suspected, even to a slight degree. Additionally, the patient had a high LRINEC score of 9. Although the LRINEC score has low sensitivity, it is advantageous due to its simplicity and ease of use. For instance, considering proactive aspiration when the LRINEC score is ≥6 (intermediate risk) might help prevent delays in diagnosis caused by underestimation of NF.

To the best of our knowledge, this is the first case report of good outcomes achieved through subcutaneous drainage alone. A PubMed search did not reveal any previous reports of percutaneous drainage for NF of the lower extremities. Cases that were cured with conservative treatment alone include those of periorbital NF [14,15], neonatal NF of the abdominal wall [16], and other special cases. Cervical NF is treated by percutaneous drainage instead of surgical debridement, typically with good results [17,18]. In cervical NF, the main purpose of this is to drain pus. A small amount of muscle may contribute to a lack of necrotic tissue in the event of myonecrosis [17]. Percutaneous drainage is less invasive than surgical debridement. It allows catheter placement with local anesthesia and a small incision, eliminating concerns about complications associated with general anesthesia. Although debridement cannot be performed, irrigation can be performed through the catheter. This approach also reduces pain and stress associated with daily wound care procedures [17]. It is unclear exactly which patients with NF will experience good outcomes with percutaneous drainage alone; however, in our cases, the subacute course and partial efficacy of the antimicrobial therapies administered may have contributed. Compared to MSSA, MRSA tends to have a more severe course, including a higher amputation rate [3,19]. MSSA is more likely to have a subacute course.

Further case series are needed to determine the indications for percutaneous drainage and whether it is as effective as surgical debridement. However, at present, percutaneous drainage should be limited to cases in which surgical debridement cannot be performed because of social or functional reasons (e.g., infected sites with significant disadvantages owing to loss of function). Surgical debridement should usually be prioritized. Based on the above considerations, if specific indications were to be proposed, they would likely include the following criteria: (i) relatively stable general condition, (ii) subacute progression, (iii) necrosis limited to the fascia, and (iv) absence of gas formation.

In Case 1, the diagnosis of NF was made using ultrasound-guided aspiration; however, surgical debridement could not be performed due to differing clinical opinions of the surgeons. To prevent such situations, it is essential to maintain good interdepartmental relationships and to establish a shared understanding of the disease. However, even with such efforts, discrepancies in diagnosis and treatment plans are common. In Case 1, the concept of subcutaneous drainage was not considered at the time of consultation with the surgeons and was later devised as a means to address the evolving clinical scenario. If subcutaneous drainage had been promptly implemented following diagnosis, the clinical course might have been more favorable. Although neither case represents the ideal management of NF, we believe that the effectiveness of subcutaneous drainage has been demonstrated.

The recommended duration of antimicrobial therapy is until surgical debridement is sufficient for infection control and clinical improvement is achieved [20]. In our two cases, antimicrobial therapy was completed, and no recurrence was observed.

## Conclusions

We encountered two cases of subacute NF of the lower extremities caused by MSSA. Diagnoses were achieved using ultrasound-guided aspiration and Gram staining. The patients were treated successfully through percutaneous drainage. The combination of ultrasound-guided aspiration and Gram staining is expected to improve diagnostic accuracy, offering a less invasive alternative than exploratory incision. Percutaneous drainage may be a less invasive alternative than surgical debridement; however, its indications require further investigation. Therefore, additional case studies are warranted.
